# Evaluation of standards for sex estimation using measurements obtained from reconstructed computed tomography images of the femur of contemporary Black South Africans

**DOI:** 10.1007/s00414-025-03430-4

**Published:** 2025-01-30

**Authors:** Oriasotie M. Ujaddughe, Jenny Haberfeld, Mubarak Ariyo Bidmos, Oladiran I. Olateju

**Affiliations:** 1 School of Anatomical Sciences, Faculty of Health Sciences, University of the Witwatersrand, Johannesburg, South Africa; 2 Anatomy Department, Ambrose Alli University, Ekpoma, Nigeria; 3 Radiology Department, Charlotte Maxeke Johannesburg Academic Hospital, Johannesburg, South Africa; 4 College of Medicine, QU Health, Qatar University, Doha, Qatar

**Keywords:** BlackSouth Africans, Femur, Computed tomography, Discriminant function analysis, Logistic regression analysis, Sex estimation

## Abstract

Dimensions of the pelvic and skull bones are known to be sexually dimorphic in various population groups. The recovery of these bones is potentially beneficial in estimating the sex in forensic cases. Since both bones are not always available for forensic analysis, standards for sex estimation must be established for other bones of the postcranial skeleton. Previous studies have reported that postcranial skeletal elements (e.g. femur) perform like the pelvis and better than the cranium in sex estimation. Thus, this study explored the potential of CT-derived femoral measurements in sex estimation in a South African population. The sample consisted of 280 contemporary Black South Africans (50% sex ratio) whose scan reports are stored in the Radiology Department of Charlotte Maxeke Johannesburg Academic Hospital, Johannesburg, South Africa. The Xiris and IntelliSpace software was used to reconstruct the images into 3D formats from which measurements were taken. Previously derived sex estimation equations of the femur were tested using data from the current study and these equations presented low average classification accuracies. Using discriminant function and logistic regression analyses, new sex estimation equations were formulated, and these new equations yielded average sex estimation classification accuracies of between 82.5 and 91.4% (by multivariable logistic regression) and 79.3–84.3% (by multivariable discriminant function). The results indicate that population-specific femoral measurements obtained by CT are more reliable than those obtained by direct osteometry and that the femur has a high sexual dimorphism with relevant forensic applications.

## Introduction

Dimension of bones of the human skeleton such as the pelvis and the skull have been shown to be sexually dimorphic in various population groups. The recovery of these bones is potentially beneficial in the estimation of the sex of the individual to which they belonged in forensic scenario cases [1–5]. However, both bones are not always available for analysis [6] and in such cases where the femur is the only preserved bone, it is still essential to estimate the sex of the individual from this bone [7]. Measurements of the femur are easy to obtain due to their linearity which also improves sex differentiation potential. Sex differentiation in the femur is due to the differences in the femoral measurements between the females and the males owing to the relatively large muscle attachments in the males [8]. Previous studies from different population groups have also shown that the femur exhibits sexual dimorphism [8–17], and the results from these studies have demonstrated similarity within the same population [8, 9, 18].

Similar studies in South Africa have also demonstrated the sex-estimation potentials of the femur [18–20]. Steyn and Iscan [18] conducted the first study of its kind in South Africa in which seven femoral and six tibial measurements from white South Africans reported sex estimation classification average accuracies that ranged from 86 to 91%. The distal breadth of the femur and the tibia were also found to be the most discriminatory [18]. In another study, the potential of the use of identification and demarking points of femoral measurements of the South African whites and blacks in sex estimation was explored by Asala [19] who found that the vertical and transverse diameters of the femoral heads were sexually dimorphic. Similarly, Asala et al. [20] subjected five measurements from the upper end and three from the lower end of the femur to discriminant function analysis and found that the vertical head diameter and medial condylar length were the most discriminatory measurements in sex estimation. Other studies on sex estimation on South African populations have demonstrated that measurements from bones of the postcranial skeleton presented with average classification accuracies that are comparable to that of the pelvis [21] and higher than that produced by the cranium [22, 23].

The equations that were formulated for sex estimation in the aforementioned studies previously conducted on South Africans produced good identification accuracies (greater than 70%). However, these studies made use of direct osteometry using the callipers. Also, the osteological collections from which the studies were carried out belong to a chronologically older population that are made up of human skeletons obtained from South Africans of European Descent (SAED) [18, 20, 24]. Furthermore, the three major South African osteological collections in Johannesburg [25], Pretoria [26], and Cape Town [27] consist of cadaveric-derived bones from bodies of those whose families could not afford the cost of burial, hence these collections have an over-representation of members of the lower socio-economic class [7, 25, 26]. It can therefore be hypothesized that the current models, functions, and equations being used for sex estimation, which were derived from measurements obtained from the bones housed in these collections may not be applicable or reliable to the contemporary population that the present study explored.

With access to modern technologies, obtaining measurements from living populations have now become possible with radiological instruments such as X-ray, Magnetic Resonance Imaging (MRI) [28, 29] and Computer Tomography (CT) [28, 30] compared to direct measurements on bones. Measurements from the CT images are more reliable and reproducible because of the high imaging resolution of the images, their sensitivity to bone, and the ease of recreating volumetric 3-dimensional versions of the CT (3-DCT) images which enables hidden structures to be easily identified and used repeatedly as a reference point for other measurements [31–36]. CT images have become more readily available because it is used for diagnostic purposes in living patients who regularly visit the hospital for various medical reasons [35]. This process continuously grows the CT records with no selection biases of individuals, individuals with socio-economic status, or individuals from specific population groups.

The sex-discriminatory ability of the femur owed to the muscle attachments at its proximal and distal ends [8] is well-documented across different population groups [8, 12, 16, 17, 20]. However, there is no report on sex estimation on the femur using CT images of the contemporary Black South African population. The selection of this population group is because it is the predominant population group in South Africa that is directly affected by highly violent crimes [37]. It is a known fact that South Africa has a high and increasing crime rate where over two million crimes are committed annually out of which about 33% are violent – leading to death [37, 38]. It is also a fact that the correct identification of an individual from bone remains in a forensic scenario is significantly aided by correctly estimating the sex of the individual which narrows the matching possibilities [39, 40] and guarantees the certainty by which sex-specific parameters like stature can be correctly estimated [1, 32, 39, 41–44]. Thus, the present study examined the CT images of the femur of the Black South African population by obtaining measurements from two- (2D) and three-dimensional (3D) reconstructed CT images. The obtained data was used to test the reliability of the current standards for the estimation of sex and to generate new models, functions, and equations for sexing the femur of the contemporary Black South African population group.

## Materials and methods

**Fig. 1:**
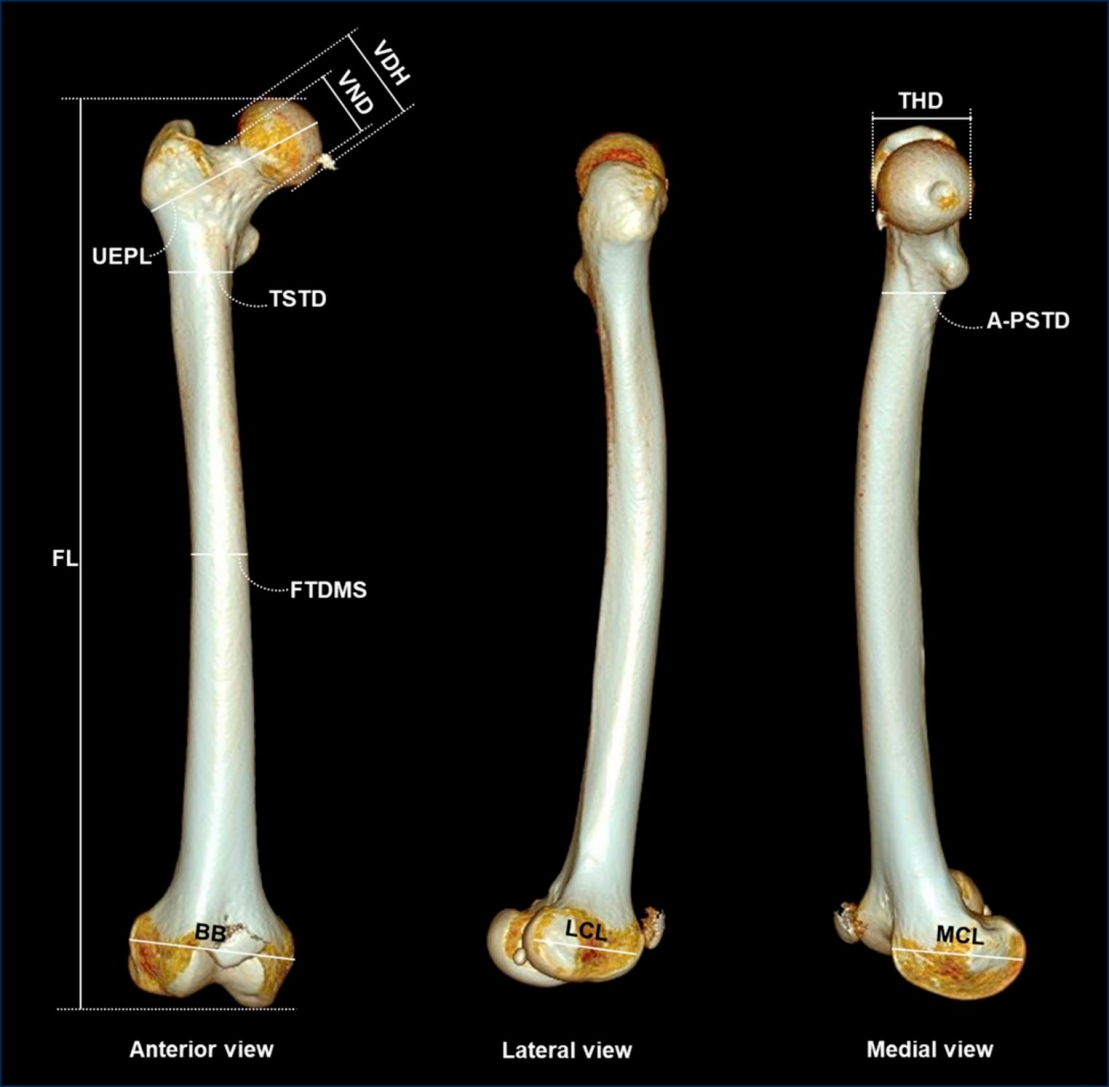
Illustrations of different views of the femur showing the selected measurements

Ethical approval (M220108) was sought and obtained from the Human Research Ethics Committee (HREC) at the University of the Witwatersrand, Johannesburg before the commencement of the present study. CT records of 280 (Female = 140; Male = 140) right femurs of Black South Africans who at the time of the scan (between January 2016 to March 2023) were between the ages of 18 and 60 years old. The scans were housed and assessed in the Radiology Department, Charlotte Maxeke Johannesburg Academic Hospital, Johannesburg (CMAHJ), South Africa. The CMAHJ is a tertiary hospital located in the Gauteng province of South Africa which serves as a referral centre for the other health facilities in and around the province. It is also one of the training institutions for the Faculty of Health Sciences of the University of the Witwatersrand, Johannesburg. These CT scans were taken using the Phillip (SOMATOM Definition AS) at a resolution of 1024 × 1024 matrix and saved in Digital Imaging and Communications in Medicine (DICOM) format. In this study, the Xiris and IntelliSpace PACS Radiology software (version 4.4.516.21) was used to reconstruct the images into 3D formats following which eleven different measurements were taken from each of the reconstructed images of the femur (Table 1; Fig. 1). Individuals whose scans exhibited signs of pathologies, deformities, or fractures were excluded from the study.

**Table 1:** A summary of the descriptions of the selected measurements taken on the femur according to previous studies [12, 17, 19, 45, 46]

Measurement	Definition
Femoral Length (FL)	Distance from the most superior point of the head of the femur to the most inferior point of the medial condyle
Transverse head diameter (THD)	Maximum diameter of the femoral head, measured on the border of the articular surface;
Femoral transverse diameter at mid-shaft (FTDMS)	Anteroposterior diameter at the midpoint of the diaphysis, at the highest elevation of the linea aspera, taken perpendicular to the ventral surface
Vertical diameter of the femoral head (VDH)	Maximum vertical distance between the upper and lower ends of the femoral head.
Upper epicondylar length (UEPL)	The linear measurement between the most superior point on the fovea capitis of the femur and the inferior aspect of the greater trochanter.
Vertical neck diameter (VND)	The minimum linear distance between the superior and inferior points on the neck of the femur.
Transverse subtrochanteric diameter (TSTD)	Transverse diameter of the proximal portion of diaphysis at a point of its greatest lateral expansion below the lesser trochanter (It is oriented parallel to the anterior surface of the femur neck)
Antero–posterior subtrochanteric diameter (A-PSTD)	Anteroposterior diameter of the proximal end of the diaphysis is measured perpendicular to the transverse diameter (it is recorded at the point of the greatest lateral expansion of the femur below the lesser trochanter and it is perpendicular to the anterior surface of the femur neck).
Bicondylar breadth (BB)	The linear distance between the femur’s medial and lateral epicondyles.
Medial condylar length (MCL)	The linear distance between the most anterior and the most posterior points on the medial condyle.
Lateral condylar length (LCL)	The linear distance on the lateral condyle is measured in an anteroposterior direction.

All measurements were taken in millimetres (mm) and were assessed for intra- and inter-observer reliability of the measuring techniques. The absolute and relative technical error of measurement (TEM and rTEM), as well as the coefficient of reliability *R*, were calculated to demonstrate the degree of measurement error with respect to the size of the measurement and the overall margin of error for each measurement in Table 2 [47–50]. After establishing the adequacy of the measuring technique, data for the calibration and validation samples were collected and analysed using IBM SPSS Statistics (version 28.01.1). Using the Kolmogorov-Smirnov and Shapiro-Wilk’s test methods, the data was tested for normality. The skewness values against the standard errors, the Q-Q plots, and histograms were all noted, and their outcomes demonstrated that the data for all measurements (except UEPL) were normally distributed (Table 3). The mean values obtained for the females and males were compared using the one-way ANOVA method and all inferences were based on a 95% confidence interval (where *p*-value ≤ 0.05 was taken as statistically significant) (Table 5).

To test the validity and reliability of four previously derived equations by Asala et al. [20] as shown in Table 6, the equations were applied to an independent sample made up of 20 females and 20 males from the present study. Subsequently, the data (*n* = 280) was subjected to univariate (univariable and multivariable) discriminant function analyses (DFA), and by applying a stepwise approach, the coefficients and constants of the best-performing sets of measurement equations were used to derive new sex estimation equations of the femur. In addition, the same data (*n* = 280) were subjected to the logistic regression analyses (LRA). To determine the sex of the different subjects and the sex classification accuracies, the values obtained in the DFA and LRA were compared with the sectioning points. In discriminating between sexes, values greater than the section point were taken as sex with a positive centroid and those less than the sectioning point were taken as the sex with a negative centroid value. With the use of the ‘leave-one-out’ classification process, the validity of the derived function equations was tested, and the original classification accuracies were noted. Also, the cross-validated values of the obtained original accuracy were recorded as shown in Tables 8, 9 and 11. The final stage of the reliability test was to apply the derived DFA equations to the independent sample made up of femoral measurements of 20 females and 20 males obtained using the same method and from the same population.

## Results

The results of the TEM, rTEM and R for the femur are shown in Table 2. The values of rTEM range between 0.80 and 4.40% for the intra-observer and 0.10–1.40% for the inter-observer. These values are within the normal limits [47–50].

**Table 2:** Measurement error (rTEM, TEM and *R*) of dimensions of the femora

	Intra-observer			Inter-observer		
Variables	TEM	rTEM	*R*	TEM	rTEM	*R*
Femoral Length (FL)	3.736	0.008	0.999	0.550	0.001	0.999
Transverse head diameter (THD)	1.546	0.034	0.999	0.660	0.014	0.999
Femoral transverse diameter at mid-shaft (FTDMS)	1.225	0.044	0.998	0.176	0.006	0.999
Vertical Diameter of the femoral head (VDH)	1.499	0.035	0.999	0.520	0.011	0.999
Upper Epiphysial Length (UEPL)	1.308	0.015	0.999	0.348	0.004	0.999
Vertical Neck Diameter (VND)	1.253	0.039	0.999	0.298	0.009	0.999
Transverse Sub-Trochanteric Diameter (TSTD)	1.156	0.033	0.999	0.265	0.007	0.999
Antero–posterior subtrochanteric diameter (A-PSTD)	0.983	0.032	0.999	0.379	0.011	0.999
Bicondylar Breadth (BB)	1.055	0.014	0.999	0.729	0.009	0.999
Medial condylar length (MCL)	1.109	0.017	0.999	0.733	0.011	0.999
Lateral condylar length (LCL)	1.081	0.018	0.999	0.479	0.007	0.999

The outcome of the normality test done using the Kolmogorov-Smirnov’s test as well as the skewness values against the standard errors showed that the set of data was normally distributed, except UEPL (Table 3).

**Table 3:** Test of normality of femoral data

	Kolmogorov-Smirnov		
Measurement	Statistics	df	p-value
THD	0.062	140	0.200
FTDMS	0.052	140	0.200
VDH	0.046	140	0.200
UEPL	0.108	140	< 0.001
VND	0.045	140	0.200
TSTD	0.047	140	0.200
APTSD	0.073	140	0.063
BB	0.069	140	0.097
MCL	0.055	140	0.200
LCL	0.056	140	0.200

df = degrees of freedom

The age distribution of the samples for both sexes is presented in Table 4. The mean age of the females was 42.61 ± 12.41 (*n* = 140) and that of the males was 38.38 ± 11.12 (*n* = 140) while the mean age of the combined sexes was 40.44 ± 11.96 (*n* = 280) (Table 4).

**Table 4:** Age distribution according to age group and sex

Age group (years)	Female (*n* = 140)	Male (*n* = 140)	Total (*n* = 280)
16–20	04	03	07
21–25	14	14	28
26–30	12	27	39
31–35	14	16	30
36–40	17	29	46
41–45	17	17	34
46–50	15	07	22
51–55	17	10	27
56–60	30	17	47
Mean ± SD	42.61 ± 12.41	38.38 ± 11.12	40.44 ± 11.96

SD = Standard deviation

The mean values of all the measurements were found to be statistically different between the two sexes where the measurements in the males were significantly higher than in the females. This result, as presented in Table 5, depicts that the femur of black South Africans is sexually dimorphic, and it is useful for the estimation of sex.

**Table 5:** Descriptive statistics of the different measurements of the femur

	Female		Male		Statistics		
Variable	Mean	SD	Mean	SD	F-Statistics	Significance	*p*-value
FL	442.19	24.26	469.63	23.56	92.17	< 0.01	< 0.001
TDH	42.42	2.92	46.90	2.70	177.66	< 0.01	< 0.001
FTDMS	26.54	2.29	28.61	2.23	58.71	< 0.01	< 0.001
VDH	42.06	2.78	46.83	3.10	183.69	< 0.01	0.002
UEPL	85.75	6.21	93.35	6.99	92.41	< 0.01	0.002
VND	29.42	2.31	32.71	2.79	115.84	< 0.01	0.002
TSTD	31.09	2.85	33.82	3.10	58.73	< 0.01	< 0.001
A-PSTD	28.31	2.80	31.35	2.77	83.71	< 0.01	< 0.001
BB	74.73	5.14	81.14	4.58	121.39	< 0.01	0.001
MCL	58.41	3.91	63.99	3.61	153.91	< 0.01	< 0.001
LCL	59.43	3.95	64.49	3.58	126.41	< 0.01	< 0.001

SD = Standard Deviation

The present study tested the reliability of the existing equations that were derived by Asala et al. [20] using data from a contemporary population. For this reason, a total of four equations from the study by Asala et al. [20] were tested on an independent sample obtained from the present study using the sectioning points from the study by Asala et al. [20]. The results of these analyses are presented in Table 6. Function 1 was the best performing as it correctly classified all the females and 50.0% of the males, with an average classification rate of 75%. Function 4 was the least performing as it correctly classified all the males but misclassified all the females, with an average accuracy of 50%. The other two equations derived by Asala et al. [20] performed like Function 1, with an average classification accuracy of 75%, correctly classifying 95.0% of the females and 55.0% of the males. This shows that most of the previous equations may still be relevant (at > 70% classification accuracy) even though they utilized the direct osteometry method on bone collections. Though correct classification rates of greater than 75% are typically accepted for sex estimation [6, 51, 52] as this provides an accuracy that is 50% greater than chance, the observation that the exiting equations are poor at classifying the males, suggest that the equation by Asala et al. [20] may not be very reliable. However, this observation in which some previously derived equations were found to have a classification accuracy of > 70% when applied to an independent sample from the current study, demonstrates that the technique used in the current study is reliable and reproducible.

**Table 6:** Classification accuracy of previously derived equations using independent samples from a contemporary black South African population for the femora

	Functions	Author	Sectioning points	Female classification (%)	Male classification (%)	Average classification (%)
Y_1_	y = (VDH × 0.176) + (UEPL × 0.080) + (VND × 0.077) + (TSTD × 0.056) + (A-PSTD × 0.010) + (− 19.346)	Asala et al. [20]	−0.225	100	50	75
Y_2_	y = (BB × 0.121) + (MCL × 0.158) + (LCL × 0.002) + (− 18.997)	Asala et al. [20]	−0.188	95	55	75
Y_3_	y = (BB × 0.121) + (MCL × 0.160) + (− 18.975)	Asala et al. [20]	−0.188	95	55	75
Y_4_	y = (VDH × 0.237) + (UEPL × 0.097) + (− 19.146)	Asala et al. [20]	−0.222	100	0	50

Y_1_ = Function 1; Y_2_ = Function 2; Y_3_ = Function 3; Y_4_ = Function 4

The results of the DFA, the sectioning points, and the unstandardized coefficients are shown in Tables 6 and 7. The univariable analyses for all the measurements showed that the original average sex estimation accuracies ranged between 65.0% (transverse sub-trochanteric diameter) and 82.9% (femoral head diameter) and on cross-validation, the classification accuracies were the same as the original classification (Table 6).

**Table 7:** Univariable discriminant function coefficient, constants and classification rates for the different measurements of the femur

			Sectioning points	Average Accuracies (%)		Male		Female	
Function	Variable	UC		OC	CV	OC	CV	OC	CV
1	THD	0.355	0.00	82.9	82.9	80	80	81.4	81.4
	(Constant)	−15.866							
2	VDH	0.34	0.00	76.4	76.4	82.1	82.1	79.3	79.3
	(Constant)	−15.099							
3	MCL	0.266	0.00	76.4	76.4	75.7	75.7	76.1	76.1
	Constant	−16.266							
4	LCL	0.265	0.00	73.6	73.7	73.6	73.6	73.6	73.6
	Constant	−16.434							
5	BB	0.205	0.00	72.1	72.1	78.6	78.6	75.4	75.4
	Constant	−16.011							
6	VND	0.39	0.00	72.1	72.1	76.4	76.4	74.3	74.3
	Constant	−12.128							
7	UEPL	0.151	0.00	71.4	71.4	77.1	77.1	74.3	74.3
	Constant	−13.553							
8	FL	0.042	0.00	70.0	70.0	72.9	72.9	71.4	71.4
	Constant	−19.065							
9	FTDMS	0.104	0.00	68.6	68.6	57.1	57.1	55.4	55.4
	Constant	−15.723							
10	A-PSTD	0.359	0.00	65.7	65.7	77.9	77.9	71.8	71.8
	Constant	−10.713							
11	TSTD	0.336	0.00	65.0	65.0	72.1	72.1	68.6	68.6
	Constant	−10.895							

UC: Unstandardised coefficient; OC: Original Classification; CV: Cross Validation. For example, Function 1 will be represented by the equation: y = (THD × 0.355) − 15.866.

The multivariable analyses of all measurements (Table 8) showed that the average original sex classification accuracies ranged between 79.3% (Function 7) and 84.3% (Function 1). Following cross-validation of the derived Functions using the leave-one-out classification method, the average classification accuracies slightly dropped to a range between 79.3% (Function 7) and 82.5% (Function 1). The equations that made use of only measurements from the proximal part of the femur produced higher classification accuracies (Function 1) when compared to those that made use of only measurements from the distal part of the femur (Function 6). The THD, VDH and VND featured prominently in the best-performing Eqs. (1–3). The average original classification for the males ranged between 80.0% (Function 7) and 85.7% (Function 1), and on cross-validation, the range changed from 79.3% (Function 6) to 82.9% (Function 1). The analyses of the data for the females yielded slightly lower sex classification accuracies with a range between 78.6% (Function 7) and 82.9% (Function 1), while on cross-validation, the range slightly changed to 73.9% (Function 7) and 82.1% (Function 1) as summarized in Table 8.

**Table 8:** Stepwise multivariable discriminant function coefficients, constants, and classification rates for the femur

				AA (%)		Female (%)		Male (%)	
Function	Variables	UC	Sectioning point	OC	CV	OC	CV	OC	CV
1	THD	0.137	0.00	84.3	82.5	82.9	82.1	85.7	82.9
	VDH	0.146							
	VND	0.078							
	TSTD	−0.017							
	A-PSTD	0.048							
	UEPL	0.015							
	Constant	−17.243							
2	THD	0.092	0.00	83.6	81.4	82.1	80.0	85.0	82.1
	VDH	0.127							
	UEPL	0.006							
	VND	0.073							
	BB	0.002							
	MCL	0.077							
	LCL	0.016							
	Constant	−18.387							
3	THD	0.151	0.00	83.2	82.1	82.9	81.4	83.6	82.9
	FTDMS	0.022							
	VDH	0.148							
	VND	0.078							
	TSTD	−0.016							
	A-PSTD	0.043							
	Constant	−17.143							
4	THD	0.111	0.00	82.1	81.4	81.4	80.7	82.9	82.1
	VDH	0.155							
	MCL	0.083							
	LCL	0.021							
	Constant	−18.193							
5	THD	0.182	0.00	81.8	81.8	82.1	82.1	81.4	81.4
	VDH	0.193							
	Constant	−16.683							
6	BB	0.071	0.00	80.0	79.6	80.0	80	80	79.3
	MCL	0.147							
	LCL	0.061							
	Constant	−18.335							
7	FL	0.006	0.00	79.3	79.3	78.6	78.6	80	80
	UEPL	0.04							
	MCL	0.148							
	LCL	0.058							
	Constant	−19.136							

AA: Average Accuracies; UC: Unstandardised coefficient; OC: Original Classification; CV: Cross Validation. For example, Function 6 will be represented by the equation: y = (BB × 0.071) + (MCL× −0.147) + (LCL × 0.061) − 18.335.

The different reliability outcomes of the derived Functions by the multivariable technique were subjected to a further assessment using independent samples of 20 females and 20 males from the present study. These Functions produced average classification accuracies ranging between 82.5% for Functions 1, 3 and 4 to 87.5% for Functions 2, 6 and 7 when applied to the independent samples (Table 9).

**Table 9:** Classification of independent sample from the same population following application of multivariable functions

Functions	Original Classification (%)	Female Classification (%)	Male Classification (%)	Average Classification (%)
2	83.6	80.0	95.0	87.5
6	80.0	90.0	85.0	87.5
7	79.3	90.0	85.0	87.5
5	81.8	75.0	95.0	85.0
1	84.3	75.0	90.0	82.5
3	83.6	75.0	90.0	82.5
4	82.1	80.0	85.0	82.5

The multivariable LRA of measured parameters of the femur bone (Table 10) showed that the average original sex classification accuracies ranged between 82.5% (Function 6) and 91.4% (Function 1). Following cross-validation of the derived Functions, the average classification accuracies slightly dropped to a range between 80.7% (Function 6), and 90.0% (Function 1). The average original classification for females ranged between 81.4% (Function 6) and 100.0% (Function 1), on cross-validation, the range changed slightly to 80.0% (Function 6) and 100.0% (Function 1). The data for males also yielded some correct sex classification with a range between 82.7% (Function 1) and 85.0% (Functions 2 and 3), while on cross-validation, the range dropped slightly to 80.6% (Function 1) and 82.1% (Functions 2 and 3). In most of the Functions generated using the DFA and LRA, some of the parameters of the femur were consistently selected such as femoral length (FL), femoral head diameter (THD), vertical diameter of the femoral head (VDH), medial condylar length (MCL), lateral condylar length (LCL), bicondylar breadth (BB) and upper epicondylar length (UEPL). It is worthy of note that these same parameters gave higher classification accuracies in the univariable analysis.

**Table 10:** Multivariate logistic regression coefficients, constants and classification rates for the femur

Variable	Unstandardized coefficient	Centroid	Sectioning point	Classification Accuracies (%)					
				Average		Females		Males	
				OC	CV	OC	CV	OC	CV
Function 1									
FL	−0.010	F = 0.915	0.007	91.4	90	100	100	82.7	80.6
THD	0.132	M = − 0.922							
FTDMS	−0.003								
VDH	−0.244								
UEPL	−0.020								
VND	−0.146								
TSDT	0.109								
A-PSTD	−0.067								
BB	−0.011								
MCL	−0.135								
LCL	0.016								
Constant	33.97								
Function 2									
THD	−0.152	F = 0.913	0	83.6	81.4	82.1	80.7	85	82.1
VDH	−0.232	M = − 0.913							
UEPL	−0.023								
VND	−0.130								
BB	0.001								
MCL	−0.130								
LCL	−0.016								
Constant	32.069								
Function 3									
THD	−0.166	F = 0.912	0	83.6	81.1	82.1	80	85	82.1
VDH	−0.234	M = − 0.912							
VND	−0.132								
BB	−0.006								
MCL	−0.130								
LCL	−0.020								
Constant	31.569								
Function 4									
FL	−0.009	F = 0.916	0	82.5	81.1	82.1	81.4	82.9	80.7
THD	−0.143	M = − 0.916							
FTDMS	0.025								
VDH	−0.236								
UEPL	−0.018								
VND	−0.129								
BB	−0.005								
MCL	−0.126								
LCL	0.008								
Constant	33.59								
Function 5									
FL	−0.009	F = 0.916	0	82.5	81.1	82.1	81.4	82.9	80.7
THD	−0.145	M = − 0.916							
VDH	−0.233								
UEPL	−0.017								
VND	−0.127								
BB	−0.001								
MCL	−0.125								
LCL	0.006								
Constant	33.616								
Function 6									
FL	−0.009	F = 0.911	0.006	82.5	80.7	81.4	80	83.5	81.3
THD	−0.138	M = − 0.917							
FTDMS	0.034								
VDH	−0.236								
UEPL	−0.018								
VND	−0.120								
A-PSTD	−0.036								
BB	−0.005								
MCL	−0.123								
LCL	0.011								
Constant	33.471								

Sectioning point = 0.00; UC: Unstandardized coefficient; OC: Original Classification; CV: Cross Validation. For example, Function 3 will be represented by the equation y = [(THD × −0.166) + (VDH × −0.234) + (VND × −0.132) + (BB × −0.006) + (MCL × −0.130) + (LCL × −0.020)] + 31.569

The different Functions derived by the multivariable LRA technique had their reliability assessed using an independent sample made up of 20 males and 20 females whose data were obtained by the same technique from the same population. These Functions produced average accuracies in the correct sex classification that ranged between 85.0% for Function 5 to 90.0% for Functions 4 as shown in Table 11. It is evident from the results that all six equations derived by the LRA were able to correctly classify 95.0% of the males.

**Table 11:** Classification of independent samples from the same population following the application of logistic regression functions

Function	Original average classification rate	Female classification (%)	Male classification (%)	Average classification (%)
4	82.5	85.0	95.0	90.0
1	91.4	80.0	95.0	87.5
2	83.6	80.0	95.0	87.5
3	83.6	80.0	95.0	87.5
6	82.5	80.0	95.0	87.5
5	82.5	75.0	95.0	85.0

## Discussion

In the practice of forensic anthropology that requires the identification of the sex of human subjects, practitioners usually prefer to use the bones of the pelvis and cranium which are widely acclaimed to have greater sexual dimorphism, unfortunately in most forensic scenarios, these two bones – pelvis and cranium may either be unavailable or when available, are badly damaged to the point that they would have lost their sex discriminatory potentials [7]. Meanwhile, the sex dimorphism prowess of the femur is considered reliable as they are usually recovered in whole or in parts. The discriminatory potentials of the epiphysis of the bone (at the proximal and distal ends) remain invaluable probably due to the muscular attachments at these ends [8]. Thus, the femur is a reliable bone for sex estimation in human identification [8, 12, 16, 17, 20].

The use of the femur in the estimation of sex has continued to gain popularity because different authors described the femur as having a considerably high forensic value (sex estimation and identity discrimination) [14, 15], and it is considered the most reliable long bone for sex and stature estimation [53]. In addition, the ability to measure bones from radiological materials e.g. CT images is now becoming popular because it allows for measurements to be obtained from contemporary populations compared to the osteological collections which are considered chronologically older and as such might not be a good representation of the recent population [14, 15, 32]. At the same time, just like some other bones, the femur exhibits distinct osteological metrics for the females and the males within the same population subgroup [7, 14, 17, 24, 42]. This is consistent with the results of this study that found significant sex differences in all the measurements (Table 5), where the measurements were significantly higher in the males than in the females owing to the stature and muscular differences between the two sexes [40, 53, 54].

This study while attempting to circumvent the drawback of obtaining data from post-mortem human parts and a chronologically older population, made use of CT images of the femora of the Black South African population which were reconstructed for accurate measurement of the bone. The results of the measurements were compared with previous sex estimation equations that utilised direct osteometric measurements on the bones of SAED. High-yielding measurements of Asala et al. [20] and Steyn & Işcan [18] were selected and the same measurements therein were utilised in this study. The findings showed that using CT-derived bone measurements is repeatable and reliable and this can be attributed to the ease of taking measurements repeatedly from the same landmark for different measurements without introducing variations. However, this greatly depends on the resolution and quality of the CT images because poor CT resolution diminished bone density, and unskilled observers will significantly affect the repeatability of the measurements [35]. This study overcame these problems by utilising subjects between the ages of 18 and 60 years who are presumed to have good bone density because bone density is known to increase by the second decade of life, peaks in the third decade and remains stable at the fourth decade before beginning to decline gradually [55]. At the same time, the observer under the guidance of a skilled radiologist was able to exclude individuals with signs of osteoporotic disorders, deformities, or fractures from the study. The repeatability of the measurements was also validated by rTEM and *R* which revealed that the measurements were within an acceptable range [47, 56]. These further showed that the measurements of the femora from the CT images were reliable and repeatable and as such suitable for obtaining measurements in real-time.

The findings of this study are consistent with previous studies that demonstrated that the linear dimensions of some long bones are sexually dimorphic, similar to other known bones like the cranium and the pelvis with such sex dimorphism [8, 12, 19, 57–59]. In this study, the discriminant function analyses, and logistic regression analyses showed a high (> 70%) classification accuracy, leading to the formulation of equations with a high sex predictive ability comparable to those formulated by previous authors [9, 12, 19]. The drawbacks of the equations of Asala et al. [20] may be that the measurements used in deriving the equations were obtained from a population of mixed ancestry like SAED [27] or from a low socio-economic class of the population like those whose families could not afford the cost of burial and had their remains donated [25]. These drawbacks were circumvented by obtaining population-specific measurements from a contemporary population using radiologic techniques (e.g. CT records) like in other reports [12, 14, 15].

The different mean values obtained for each of the measurements in this study were compared with the measurements by Asala et al. [20] (Table 12). The Transverse diameter of the head (TDH) and the femoral transverse diameter of the midshaft (FTDMS) for this study are within the same range as that of Asala et al. [20]. Overall, the differences in the average mean values for all the measurements when those from this study were compared with that of Asala et al. [20], ranged between ± 6.99 mm for females and ± 5.35 mm for males. The greatest variations were obtained from the comparison between the dimensions of TSTD and A-PSTD of this study and Asala et al. [20]. Therefore, the outcomes of the comparisons of the mean dimension and the observed similarities suggest that the different dimensions obtained by the previous authors, from this same population, may be similar, but with some observed differences which are enough to account for the lower classification accuracies obtained for the existing equations. This is demonstrated by the classification accuracies of between 50.0% and 75% obtained following the validity tests that have been done with the existing equations derived by Asala et al. [20] using the independent samples (Table 6). The observed similarities, however, confirm that the existing data may not be reliable to a great degree of certainty, and that the study technique deployed in the current study is reliable and reproducible.

**Table 12:** Comparison of mean measurements of the femur from a previous study [20] and the current study

Variable	Current study		Asala et al. [20]	
Sex	Female	Male	Female	Male
FL	442.19 ± 24.26	469.63 ± 23.56		
TDH	42.42 ± 2.92	46.90 ± 2.70		
FTDMS	26.54 ± 2.29	28.61 ± 2.23		
VDH	42.06 ± 2.78	46.83 ± 3.10	40.7 ± 2.32	45.4 ± 2.55
UEPL	85.75 ± 6.21	93.35 ± 6.99	85.4 ± 4.62	95.0 ± 5.52
VND	29.42 ± 2.31	32.71 ± 2.79	28.8 ± 1.91	32.3 ± 2.28
TSTD	31.09 ± 2.85	33.82 ± 3.10	24.1 ± 1.95	31.8 ± 2.56
A-PSTD	28.31 ± 2.80	31.35 ± 2.77	24.1 ± 2.03	26.0 ± 2.13
BB	74.73 ± 5.14	81.14 ± 4.58	71.8 ± 4.88	78.9 ± 3.75
MCL	58.41 ± 3.91	63.99 ± 3.61	57.6 ± 3.41	63.7 ± 3.68
LCL	59.43 ± 3.95	64.49 ± 3.58	58.9 ± 3.33	64.0 ± 3.17

The outcomes of this study showed that the femora of Black South Africans are sex dimorphic, evidenced by the statistically significant sex differences in all the measurements (Table 5). This finding is consistent with the previous studies that presented the femur as a useful tool for sex estimation [18, 19]. The transverse diameter of the head and the vertical diameter of the head were found to be the most sex-discriminatory measurements (Table 7). The measurements that are associated with the proximal end of the femur like THD, VDH, VND, TSTD, A-PSTD, and UEPL in a multivariable analysis, were more discriminatory (Function 1 in Table 8) than those that are associated with the distal end of the femur such as BB, MCL, and LCL (Function 6 in Table 8) and are also similar to previous studies by Steyn & Işcan [18] and Curate et al. [49] who reported that the vertical neck diameter and proximal dimensions of the femora are the most predictive measurements for sex discrimination. The obtained classification accuracies of 79.3 – 84.3% using DFA (Table 8) and 82.5–91.4% using LRA (Table 10) show that the use of postcranial skeletal elements outperform the DFA outcome for cranium [25] and LRA outcome for cranium [22]. However, the results of these analyses (DFA and LRA) in the current study are comparable to the performance of the DFA of pelvic measurements [7, 60] which agrees with the observations made by Krüger et al. [22] and Liebenberg et al. [23].

When the existing equations by Asala et al. [20] were applied to the independent sample, 75% of the equations performed well with over 70% classification accuracies (Table 6). This is comparably lower when compared with those of this study which resulted in over 75% classification accuracy when the DFA and LRA equations were applied to independent samples (Tables 9 and 11). This further indicates that the previous equations may not be very reliable and applicable to the current population for sex estimation, however, the present results showed that population-specific femoral measurements obtained by CT are more reliable than those obtained by direct osteometry from the same, but a chronologically older population group. It also further shows that the CT approach for obtaining measurements from bones is accurate and reliable. Interestingly, the equation y= [(VDH × 0.237) + (UEPL × 0.097) − 19.147] derived by Asala et al. [20] produced a poor classification rate of 50% (Table 6). This was unexpected because VDH and UEPL which are in the equation have mean measurement values that are within the same range as the derived equations of this study (Table 12). Unfortunately, this study could not ascertain the reasons for the observed differences.

The results of the LRA in the present study demonstrated a pattern that is similar to the results of the DFA. Femoral measurements were consistently selected in formulating high-performing equations of both DFA and LRA, these include FL, TDH, VDH, MCL, LCL, BB and UEPL. It is important to state that these measurements produced higher classification accuracies when the data were subjected to univariable analysis. They would probably have been selected due to their sex-prediction abilities (Table 7). Following the application of the Functions generated using the LRA to the independent samples, it was better at classifying males (Table 11) like that of the DFA which was better at classifying males (Table 9). The Functions generated using LRA had a greater ability to classify sex than that which resulted from the application of DFA on the independent samples (Tables 9 and 11). Functions with a higher number of variables were found to also have higher classification accuracies for both the LRA and DFA. However, Functions with fewer variables, yet presenting with > 80% classification accuracies will be more useful in forensic applications because in some forensic scenarios, the femur may be recovered in part, thus only a few measurements may be obtainable from the fragments.

The equations generated in this study from a combination of different femoral measurements using DFA and LRA, resulted in average classification accuracies that are greater than 80%. These are important observations because, in forensic studies, a classification accuracy greater than 80% is generally considered acceptable [6, 51, 52]. Also, in the present study, the drop in Cross Validation is between 0 and 2.5%, which is good because cross-validated classification accuracy values with a drop of less than 5% when compared to the original classification value are also generally considered acceptable [5]. Based on the higher LRA classification accuracies (> 80%) obtained in the present study and the fact that LRA produced better identification accuracies when applied to the independent samples (Tables 9 and 11), it is thus suggested that LRA is a superior tool for metric sex-estimation technique than the DFA similar to other reports [5, 61, 62].

The current study has shown to be more promising in sex estimation and thus validates the assumption that the data from a contemporary population are different from that of the chronologically older population. This difference may be due to improvements in socio-economic circumstances of the recent population, improved standards of living, and advancements in healthcare [63] which may account for changes in the skeletal structure of individuals over time, even within a defined population. Also, the difference can be due to improvements in anthropological methods such as use of non-invasive techniques which permits measurements from a living population [42, 63–65] as against the traditional direct osteometric method [20]. These changes may affect the applicability of previously derived equations and models for sex estimation from these chronologically older samples to make forensic conclusions on contemporary populations [66].

## Conclusion

Sex estimation is useful in identifying individuals including the black South African population. The use of metric analysis for sex estimation is limited by population-specificity, thus underscoring the need to obtain measurements from a defined population such as black South Africans. In doing so, the place of contemporaneous and population-specific data obtained from bones that demonstrate sexual dimorphism is invaluable. The use of radiological techniques like the CT provides a means of sampling the living population which is beneficial for anthropology. The CT has also proven to be very useful in obtaining measurements on bones because the method provides the practitioner with the advantage of obtaining accurate and easily reproducible sets of measurements which has the potential for better reliability. The high average classification accuracies which range from 79.30 to 84.30% found in the femur show that this bone has a high potential for sex discrimination (estimation) and as such very relevant to forensic application. However, the shortfall of this study is the inability to determine the specific sub-population groups within the South African black population that was used for this study. To properly profile the South African Black population along its different sub-population groups, it may be beneficial in the future to design and implement a study of this manner as a prospective study. This will enable a thorough and fit-for-purpose biodata in real time which may therefore guarantee more defined demographic data for delineating the different sub-population groups.

## Data Availability

Data are available on request.
